# Datasets for Automated Affect and Emotion Recognition from Cardiovascular Signals Using Artificial Intelligence— A Systematic Review

**DOI:** 10.3390/s22072538

**Published:** 2022-03-25

**Authors:** Paweł Jemioło, Dawid Storman, Maria Mamica, Mateusz Szymkowski, Wioletta Żabicka, Magdalena Wojtaszek-Główka, Antoni Ligęza

**Affiliations:** 1 AGH University of Science and Technology, Faculty of Electrical Engineering, Automatics, Computer Science and Biomedical Engineering, al. A. Mickiewicza 30, 30-059 Krakow, Poland; maria.anna.mamica@gmail.com (M.M.); mateuszszymkowski97@gmail.com (M.S.); 2 Chair of Epidemiology and Preventive Medicine, Department of Hygiene and Dietetics, Jagiellonian University Medical College, ul. M. Kopernika 7, 31-034 Krakow, Poland; dawid.storman@doctoral.uj.edu.pl; 3 Students’ Scientific Research Group of Systematic Reviews, Jagiellonian University Medical College, ul. M. Kopernika 7, 31-034 Krakow, Poland; wiola.zabicka@gmail.com (W.Ż.); wojtaszekglowka@gmail.com (M.W.-G.)

**Keywords:** systematic review, cardiovascular, artificial intelligence, dataset, automated emotion recognition, automated affect recognition, affective computing

## Abstract

**Simple Summary:**

We reviewed the literature on the publicly available datasets used to automatically recognise emotion and affect using artificial intelligence (AI) techniques. We were particularly interested in databases with cardiovascular (CV) data. Additionally, we assessed the quality of the included papers. We searched the sources until 31 August 2020. Each step of identification was carried out independently by two reviewers to maintain the credibility of our review. In case of disagreement, we discussed them. Each action was first planned and described in a protocol that we posted on the Open Science Framework (OSF) platform. We selected 18 works focused on providing datasets of CV signals for automated affect and emotion recognition. In total, data for 812 participants aged 17 to 47 were analysed. The most frequently recorded signal was electrocardiography. The authors most often used video stimulation. Noticeably, we did not find much necessary information in many of the works, resulting in mainly low quality among included papers. Researchers in this field should focus more on how they carry out experiments.

**Abstract:**

Our review aimed to assess the current state and quality of publicly available datasets used for automated affect and emotion recognition (AAER) with artificial intelligence (AI), and emphasising cardiovascular (CV) signals. The quality of such datasets is essential to create replicable systems for future work to grow. We investigated nine sources up to 31 August 2020, using a developed search strategy, including studies considering the use of AI in AAER based on CV signals. Two independent reviewers performed the screening of identified records, full-text assessment, data extraction, and credibility. All discrepancies were resolved by discussion. We descriptively synthesised the results and assessed their credibility. The protocol was registered on the Open Science Framework (OSF) platform. Eighteen records out of 195 were selected from 4649 records, focusing on datasets containing CV signals for AAER. Included papers analysed and shared data of 812 participants aged 17 to 47. Electrocardiography was the most explored signal (83.33% of datasets). Authors utilised video stimulation most frequently (52.38% of experiments). Despite these results, much information was not reported by researchers. The quality of the analysed papers was mainly low. Researchers in the field should concentrate more on methodology.

## 1. Introduction

Facilitating access to databases seems to be an essential matter in the field of machine learning (ML). Publicly available, reliable datasets could drive research forward, making it unnecessary to re-run similar yet complicated experiments in order to obtain sufficient data. Credible work relies on proper arrangement, validation, adjustment, and fairness in artificial intelligence (AI) [1,2].

Moreover, sufficient descriptions of the scientific methods in AI are a constant challenge. It seems to be particularly valid in automated affect and emotion recognition (AAER) studies, which fall under the field of human–computer interaction (HCI), linking psychology, computer science. and biomedical engineering. As human emotions affect multiple channels, research on this topic is being conducted based on speech, facial expressions, gestures or physiological signals, which became exceptionally popular in the last decade [3].

Increasing interest in the field, among others, comes from broad application prospects. Recent studies point out the potential usage of emotion recognition techniques in medical fields, public security, traffic safety, housekeeping, and related service fields [4].

The topic is extensive, as it covers both data acquisition and computation. A typical experiment in AAER involves several steps [5]. Firstly, the researchers need to adopt a specific perspective on the field, as many exist that consider the universality [6,7] of emotions or their structure [8]. The theoretical approach imposes an understanding of emotions, selections of material used for stimulation, and interpretations. However, the general structure of elicitation experiments that are carried out to gather the data from human participants remains stable [9]. To evoke emotions, passive (e.g., video, music, or pictures presentation) or active stimulation (e.g., game playing, interaction with virtual reality, or conversation) is used [5]. Eliciting material may have different lengths, types, and quantities. After the stimulation phase, the subjects are asked how they felt. Several validated instruments enable it, e.g., Self-Assessment Manikin (SAM) [10].

During the stimulation phase, subjects are connected to measuring devices. Researchers use dedicated hardware [11,12,13] and experiment with smartphone [14] or wearable [15,16] technologies, especially with CV signals [17,18,19]. Among others, gathered data include physiological signals [20] (e.g., heart, skin, brain, respiratory system, and eye work), facial expressions [21], and speech [22,23]. Typically, several signals are collected in order to improve the accuracy of the AI system used for AAER [24].

Next, the recognition phase begins. It involves data preprocessing, classification or regression, and finally, validation [5]. Due to its flexibility resulting in, e.g., reduced data preprocessing time [5], deep learning (DL) techniques are widely adopted [25,26,27], along with classical approaches in AAER [28,29,30,31].

As the data collection process in experiments within this field is complex and multi-stage, the problems may occur on many levels. It is thus crucial to plan the experiment and report upon it in adequate detail [32].

The replicability crisis in both psychology and computer science also affects studies on AAER [5,33,34]. Poor methodological conduct often makes it impossible for existing research to be replicated or reproduced. Even in renowned and well-established research that dictates the social order, the phenomenon is widely present [35,36].

Datasets collected inadequately might contribute to lowering the credibility of emerging research (influencing model development by introducing undesirable biases) and waste of time and resources. This issue has been widely discussed before and is known as the *garbage in, garbage out problem* [37,38]. Avoiding bias and proper validation of experiments are crucial to eliminating it [32].

Promisingly, publishing source codes and data is becoming a desirable standard in computer science [39,40,41,42]. Journal initiatives [43,44] on the topic emphasise the importance of computational research reproducibility and promote open research. In turn, preregistration of the research plan, taking into account the hypotheses and defining step-by-step the methodology allows for improving the quality of the research and its reproducibility from a psychological perspective [45,46].

To create a reliable model presenting a high degree of emotion or affect recognition precision, it is relevant to limit external and internal factors potentially confounding the collected measurements [47,48].

The confounding effect of incomplete control may arise from any stage of the study. For instance, subjects with somatic disorders might affect measures of features, mood disorders, or alexithymia, which is estimated to affect 13% of the population [49].

Each stage of an experiment leading to AAER should be repeatable and standardised among subjects. AAER concerns stimuli presentation, assessment of elicited emotions by the subject, collection of physiological parameters, and laboratory environment, including the presence of experimenter and individual factors [5].

While measuring emotional and affective responses in the laboratory environment using objective methods reduces the risk of self-reported bias, the risk of contextual non-integrity remains. This creates the need to document all the contextual environmental aspects that could influence the measurement [50].

Along with the pervasiveness of wearable devices available to register user psychological parameters during daily activities, AAER is reached [51,52]. Wearable devices are proven to measure efficiently CV signals while being offered at low prices [53,54]. However, the challenge remains to design credible ML models able to deal with the broad spectrum of possible emotions and lack of universality in this category among cultures [7].

Studies on ubiquitous computing are growing in number [55,56,57]. Due to the constraints of time and human resources, all these results could not be read. Therefore, creating summaries along with the analysis of evidence is now necessary [58]. Describing the data together with a critical appraisal helps to determine, for example, the actual accuracy of the methods and to highlight those articles whose results are derived from a high-quality methodological process. The selection of studies answering a similar research question may be chaotic, purposeful, or systematic [59]. The latter method reduces the risk of researchers steering conclusions, as it follows restrictive, transparent criteria [32,60,61].

Because of the above and since previous similar studies on AAER were of weak reliability [5], we decided to present a systematic review on the topic, corresponding to approved standards, to limit the risk of bias (RoB). We review public datasets available for AAER with the use of AI, utilising physiological modalities as an input with the focus on CV signals.

This paper is a part of the project on a systematic review of studies focused on AAER from CV signals with AI methods. For more details, see the protocol [62] and our previous conference paper [63].

### Research Questions

1. What are the datasets used for AAER from CV signals with AI techniques?
2. What are the CV signals most often gathered in datasets for AAER?
3. What were other signals are collected in analysed papers?
4. What are the characteristics of the population in included studies?
5. What instruments were used to assess emotion and affect in included papers?
6. What confounders were taken into account in analysed papers?
7. What devices were used to collect the signals in included studies?
8. What stimuli are most often used for preparing datasets for AAER from CV signals?
9. What are the characteristics of investigated stimuli?
10. What is the credibility of included studies?

## 2. Methods

### 2.1. Eligibility Criteria, Protocol

Papers in which more than half of the sample constitutes a specific population (e.g., children or people with illness) were excluded. All experiments needed to be carried out in laboratory settings. We considered any type of publication to be eligible in which CV signals and AI methods were used for AAER. The primary focus of our whole project [63] was the performance of these computer programs (e.g., specificity, sensitivity, accuracy). For this focused systematic review, we imposed additional inclusion criteria, namely public availability of the data.

Due to double referencing, some of the references were overlapping. These were post-conference books and full proceedings. We excluded them as they contained little information about specific chapters. Nevertheless, we did not reject these particular sections. We excluded introductions to Special Issues in a journal or section, letters to editors, reviews, post-conference books, full proceedings (but not qualified papers), and case studies.

The review protocol was published on the Open Science Framework (OSF) [64] and then registered there [62] on 18 March 2021. All additional information about methods can be found in the protocol.

### 2.2. Search Methods

We searched article databases (MEDLINE, Web of Science, dblp, EMBASE, Scopus, IEEE, Cochrane Library) and preprint databases (medRxiv, arXiv). The complete search was done on 31 August 2020.

To develop the MEDLINE strategy (see protocol on OSF [62]), we combined MeSH (controlled vocabulary) and free-text words related to AAER, CV signals, and AI. Then, these strings were translated for other sources utilised in the search. We adopted no date or language restrictions.

Additionally, we screened full texts of included papers for otherwise not identified studies. We included them in further steps of identification.

### 2.3. Definitions

We used the following definitions. AAER [65,66] refers to finding patterns with specific signals (e.g., behavioural, physiological) consistent with detected states. AI refers to software able to perform tasks as accurately as intelligent beings (e.g., humans) [67]. DL refers to the architecture of neural networks comprising at least two hidden layers [68]. Performance metrics, which refer to a mathematical evaluation of model predictions with ground truth [69]. CV signals refer to an electrocardiogram (ECG), pulse oximetry (POX), heart rate (HR), intracranial pressure (ICP), pulse pressure variation (PPV), heart rate variability (HRV), photoplethysmogram (PPG), blood volume pressure (BVP), and arterial blood pressure (ABP) [53,70].

### 2.4. Data Collection

EndNote (Claritive Analytics^®^) and Rayyan [71] were utilised for deduplication of identified references. P.J., D.S., M.S., and M.M. used the Rayyan [71] application to screen the remaining references independently. Subsequently, full texts were assessed separately by P.J., D.S., M.S., and M.M. for meeting inclusion criteria.

P.J., D.S., M.S., M.M., W.Ż., and M.W.G. collected all necessary data independently using a pre-specified extraction form. We gathered bibliographic data (e.g., year, journal name) and information about authors, funding, and conflicts of interest. We also focused on population, models, and outcomes—AI methods and additional analyses, e.g., interpretability, as specified in the protocol (see OSF [62]).

Pilot exercises were conducted before each phase, namely screening of abstracts and titles, full text evaluation, and extraction of the data. By doing so, we aimed at improving the sense of understanding among the reviewers. When discrepancies occurred (at each step of data identification), they were resolved via discussion.

### 2.5. Quality Assessment

The methodological credibility of included studies was assessed using a tool developed by our team (see Appendix C). The method was based on well-grounded techniques, namely Quality Assessment of Diagnostic Accuracy Studies (QUADAS) [72], Prediction model Risk Of Bias ASsessment Tool (PROBAST) [73], and an instrument provided by Benton et al. [74] as it was dedicated to the same study design as included by us. The process of evaluation was preceded by pilot exercises. We rated RoB independently in pairs (P.J., D.S., M.S., M.M., W.Ż., and M.W.G.). Discussion resolved all discrepancies.

The utilised tool constituted of eight questions (items):

1. Was the sample size pre-specified?
2. Were eligibility criteria for the experiment provided?
3. Were all inclusions and exclusions of the study participants appropriate?
4. Was the measurement of the exposition clearly stated?
5. Was the measurement of the outcome clearly stated?
6. Did all participants receive a reference standard?
7. Did participants receive the same reference standard?
8. Were the confounders measured?

Items were assessed using a three-point scale with the following answers: yes/partial yes, no/partial no, and not reported resulting in high, low, or unclear RoB. For more details, see Appendix C.

### 2.6. Analyses

We concentrate on descriptive synthesis regarding characteristics of populations and collected datasets, i.e., stimuli, signals, devices, emotions, and affect. We also present results regarding credibility.

The quantitative summary with sensitivity, heterogeneity, and subgroup analysis of all papers is not the purpose of this focused review. For more details, please refer to the protocol [62] and other papers from the project [63].

## 3. Results

From 4649 records, we identified 195 studies that met our eligibility criteria. Then, we selected a sub-sample of 18 papers. Each paper provides one validated, publicly available dataset, including CV signals with labels regarding emotions or affect.

Names of datasets described in included papers are as follows: Database for Emotion Analysis using Physiological signals (DEAP) [75]; Multimodal Analysis of Human NOnverbal Behaviour in real-world settings–Human-Computer Interaction (MAHNOB-HCI) [76]; MEG-based multimodal database for DECoding AFfective physiological responses (DECAF) [77]; a dataset for Affect, personality and Mood research on Individuals and GrOupS (AMIGOS) [78]; a multimodal databASe for impliCit pERsonaliTy and Affect recognitIoN using commercial physiological sensors (ASCERTAIN) [79]; AUgsburg Database of Biosignal 4 (AuDB-4) [80]; Emotion Recognition Smartwatch (ERS) [81]; IT Multimodal Dataset for Emotion Recognition (ITMDER) [82]; Database for Affective Gaming (DAG) [83]; Quality Adaptive Multimodal AFfect recognition system for user-centric multimedia indexing (QAMAF) [84]; Virtual Reality Affective Dataset (VRAD) [85]; NEME [86]; WEarable Stress and Affect Detection (WESAD) [87]; a Multi-modal Physiological Emotion Database for discrete emotion recognition (MPED) [88]; database of multimodal (Face, Body gesture, Voice and Physiological signals) recordings (emoFBVP) [89]; a database for emotion recognition through EEG and ECG signals from wireless low-cost off-the-shelf devices DREAMER [90]; Multi-subject Affective Physiological Database (MAPD) [91]; and Mazeball Dataset (MD) [92].

**Figure 1:**
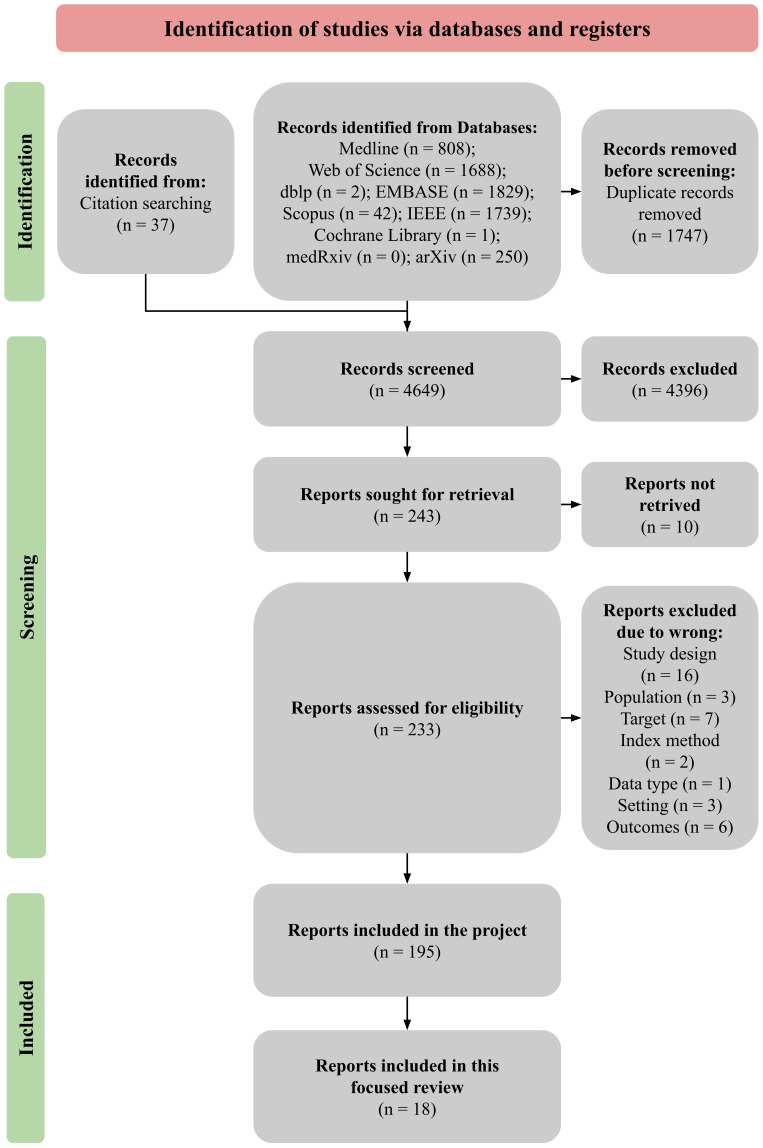
Preferred Reporting Items for Systematic Reviews and Meta-analyses (PRISMA) study flow diagram [63].

Supplementary File S1 (OSF [64]) and Appendix A and Appendix B contain the list of all included studies, the subgroup of datasets analysed in this review, and the excluded studies with reasons, respectively. The remaining included studies are considered in other articles from the project [63]. The flow of our study is presented in Figure 1. Our reporting is consistent with Preferred Reporting Items for Systematic Reviews and Meta-analyses (PRISMA) guidelines with diagnostic test accuracy (DTA) extension [93].

### 3.1. Included Studies

Included studies were published mainly in scientific journals (66.67% of papers, mean Impact Factor = 7.01) [75,76,77,78,79,80,81,85,86,88,90,92]. The most popular was *IEEE Transactions on Affective Computing*. Out of all the authors, 27.78% did not report on funding [77,82,87,89,91], while 88.89% did not inform about competing interests [75,76,77,78,79,80,82,83,84,86,87,88,89,90,91,92].

None of the studies provided source code of executed analyses, while only one study (5.56%) reported registering protocol [92].

### 3.2. Experiments

The total number of elicitation experiments was 21, presented in 18 papers. Three of the studies (16.67%) carried out two trials each [77,78,81]. It was found that 76.19% of experiments were conducted using passive stimulation solely [75,76,77,78,79,80,81,82,84,86,88,90,91], while 19.05% used only active elicitation (e.g., video games) [83,85,89,92]. One experiment (described in Schmidt et al.’s paper [87]) used both passive (video) and active stimulation (meditation and Trier Social Stress Test (TSST). The essential characteristics of experiments regarding stimuli are presented in Table 1.

Most of the experiments did not use stimuli from validated databases (71.43%, e.g., FIFA 2016, YouTube videos) [75,76,78,80,81,83,84,85,86,87,88,89,91,92], whereas public sources (e.g., DEAP, DECAF) accounted for 23.81% [77,79,81,90]. Pinto [82] used both forms. In 47.62% of experiments, the justification for the choice of the database was not reported [77,79,81,83,84,86,87,89,91,92]. Pinto [82] partially reported on it. In 42.85% of experiments, validation was provided by conducting a pilot study or preliminary classification by researchers [75,76,77,78,81,85,88,90]. Stimuli were described by authors most frequently in terms of valence (52.38% of experiments) [75,76,77,78,80,82,85,86,90,92], arousal (52.38%) [75,76,77,78,80,82,85,86,90,92], and discrete emotional tags (38.10%) [76,77,81,88,89,91]. Four experiments [77,79,83,84] did not report on it at all.

The presence of diseases or disorders was the most often controlled factor in participants (61.90% of experiments) [75,76,78,81,82,85,86,87,88,90,91]. The mood was controlled using the Positive and Negative Affect Schedule (PANAS) tool in two experiments (9.52%) [78]. In only one experiment, the authors checked if the participants were able to recognise emotions or affective states correctly [85]. In examining factors controlled in the laboratory, the most frequent was found to be brightness (33.33% of experiments) [75,77,78,90,91], followed by volume (28.57%) [78,80,82,84,91], presentation of stimuli (14.29%) [75,80,85], the comfort of participants (9.52%) [77,86], and time of the day (9.52%) [81]. In 23.81% of experiments, it was not reported which factors were controlled [77,80,83,84,92]. Additionally, in four experiments (19.05%), personality was measured in participants using the Big Five Personality Test [78,79] or the Eysenck Personality Questionnaire (EPQ) [91].

In the assessment of emotions and affect by participants, the most prevalent instruments used were: SAM (38.10% of experiments) [75,78,82,85,87,88,90] for valence, arousal and dominance, selecting a discrete emotion from the provided list (23.81%) [76,78,83,89], and PANAS (19.08%) [81,87,91].

### 3.3. Signals and Devices

Table 2 summarises applied devices and recorded CV signals. Apart from CV signals, electrodermal activity (EDA) [75,76,78,79,80,82,83,84,87,88,91,92] is available in 66.67% of datasets. Thus, these are the most prevalent data. The next most common are face video [75,76,77,78,79,83,84,89,91], electroencephalography (EEG) [75,76,78,79,84,85,88,90], respiration [75,76,80,82,83,88], and electromyography (EMG) [75,77,80,83,87] in 50%, 44.44%, 33.33%, and 27.78% of datasets, respectively. The remaining signals include, e.g., magnetoencephalography (MEG), gyroscope, accelerometer, and audio. There is only one dataset that focuses solely on CV signals [86]. The authors recognise devices used for recording CV signals as wearable in 38.89% of studies [78,79,84,85,87,89,90].

### 3.4. Validation

As we included only validated datasets in this analysis, all of the papers explored AAER with AI. Out of all the papers, 55.56% [75,76,77,78,82,85,86,90,91,92] conducted experiments with only one type of ML algorithm, while the rest explored more methods. In total, the data were validated using AI methods 33 times. Support vector machine was used most frequently (33.33%) [76,77,79,82,83,84,85,86,89,90,91]. Naive Bayes (NB) [75,78,79,84], random forest (RF) [81,83,87,91], and DL [88,89] were the second most explored techniques (12.12% each).

The authors classified 61 different discrete states in total. The most commonly classified one was sadness [81,86,89,91], occurring in 22.22% of papers. The following states were examined in two papers each (11.11%): fear [89,91], anger [89,91], amusement [87,91], anxiety [89,92], boredom [89,92], happiness [81,89], and neutral state [88,89]. Additionally, the authors used affect space in 12 (66.67%) papers [75,76,77,78,79,80,82,83,84,85,86,90].

All of the datasets were validated in classification experiments. Authors of only two datasets (11.11%) [86,89] compared their results with other publicly available data.

### 3.5. Population

The total number of analysed people was 916, with a mean number of 43.62 participants and a range from 3 [80] to 250 [91]. However, due to, e.g., missing data, the datasets contain complete information for only 812 of them.

The remaining characteristics of the population are shown in Table 3. five experiments (23.81%) were approved by the ethics committee [81,85,88,90]. Participant consent was obtained in 15 experiments (71.43%) [75,76,77,78,79,82,85,86,87,88,89,90,91]. Only one experiment ensured the privacy (by anonymisation) of participants [90].

### 3.6. Credibility

The general RoB was analysed in two scenarios—with or without the first item of the proposed tool (see Section 2.5). We excluded the first question in the second condition because none of the included studies reported on pre-specification of sample size.

**Figure 2:**
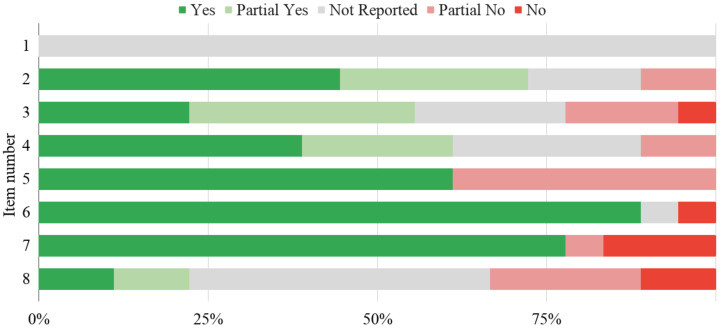
Risk of bias (RoB) in included studies.

Of all the studies, 77.78% were of low quality in both scenarios, whereas 22.22% [78,85,86,90] and 11.11% [86,90] were of unclear quality in the first and second conditions, respectively. Two studies [78,85] were of high quality according to the latter scenario. The RoB across all RoB items is presented in Figure 2. The reference standard was provided for all participants in the same way in 16 studies (88.89%) [75,76,77,78,79,81,82,83,84,85,86,87,88,89,90,91]. For participants from 14 studies (77.78%) [75,76,77,78,79,81,82,83,84,85,86,89,90,91], the same reference standard was given. Thirteen studies (72.22%) [75,76,78,79,81,82,85,86,87,88,89,90,92] did not show any flaws in terms of providing eligibility criteria for the experiment. These are the most satisfied questions.

However, 14 papers (77.78%) [75,76,77,79,81,82,83,84,86,87,89,90,91,92] did not control confounders or did not report it. Measurement of exposition [80,83,84,86,87,89,91] and outcomes [76,77,79,80,83,84,89] was flawed, or authors did not mention it, in seven studies (38.89%).

All ratings are presented in Table 4. Among them, the most frequent was *yes*, marked in 43.06% of cases. However, the second most prevalent was *not reported*, which was assessed 27.08% times.

### 3.7. Additional Analyses

Please refer to the protocol [62] and our other papers [63] from the project on AAER from CV signals with AI methods for additional analyses.

## 4. Discussion

The paper search conducted in this study revealed that there are 18 publicly available validated datasets for AAER from CV signals. The methodological credibility assessment showed that only two studies are of high quality, suggesting a significant need for developing good scientific practices.

Furthermore, none of the studies provided a source code used for the validation experiments. It opens a discussion on replicability, which we are witnessing in science nowadays [5]. Experiments in included papers were conducted on small samples. The number of participants exceeded one hundred only in one study.

What is more, the subjects’ background information was poorly described. Only four studies established that the participants were either Chinese or European. According to Wierzbicka [94,95], the history behind a person (and the language he or she speaks) may play a crucial role in the emotional states they experience and thus should be controlled. Feldman later disseminated this belief in her approach [7].

Another bothering aspect of the analysis is that an ethical commission approved experiments described by only four papers, and only one study mentioned ensuring the privacy of participants. It lights up red flags in terms of maintaining ethical standards or suggests negligence of reporting crucial information. Authors of experimental studies should more carefully examine this aspect.

Additionally, the authors either selectively controlled the influence of potential confounders or did not do so at all. Various CV diseases, mental disorders [49], and participants’ moods and personalities may affect AAER from physiological signals [78]. Therefore, we believe authors should include such information.

The problem in assessing quality in systematic reviews is about distinguishing how much the authors did not take care of the methodological regime and how much they did not report the details of the research process [60]. Therefore, it is recommended that when submitting an article to the journals’ editorial office, the authors fill in a checklist and mark the exact place where they have included the minimum necessary descriptions of the operation process [32,96].

On the other hand, we observed great diversity in the choice of physiological signals, stimuli type and length. What is more, 38.89% of the studies used wearable devices to perform measurements. Considering the increasing popularity and facility of these instruments [78], it gives the excellent potential for future adoption of proposed methods in real-life scenarios. Thanks to recent advances in the field of sensors technology, such devices are well-suited for daily usage. They do not require complicated instalments, are comfortable to wear, and are easy to use [97]. However, one should remember that there are still many limitations standing in the wy of the wider use of wearable devices in AAER. First of all, the quality of physiological signals is still noticeably lower than that of medical-level equipment [98]. What is more, the data gathered by such instruments in non-laboratory settings are often flawed, with noise coming from motion or misplacement [99].

Similarly to our study, the CV databases were also explored by Merone et al. [100]. The authors investigated 12 datasets with the inclusion criteria of having an ECG signal. In addition, they analysed included sets in terms of many parameters, e.g., the number of ECG channels and electrodes type. However, they did not primarily focus on emotions or affect. They included only one paper [101] covering this scope, which we did not consider eligible for inclusion as it did not meet the criteria. Since datasets including CV signals are still unexplored, we cannot discuss our results with other authors. Furthermore, Hong et al. [102] analysed ECG data systematically using DL. Still, they identified only one study about AAER [103], but it was not in their primary interest, so they did not describe it in detail.

However, in the literature, there are plenty of reviews (systematic and not) focusing on AAER from multiple signals or focusing on specific ones, e.g., EEG [104,105,106], or covering multiple modalities [53,107,108,109,110,111]. Still, their quality has been thoroughly criticised in our recently published umbrella review [5].

In line with these results, in the current literature, we found a shortage of highly credible and methodologically reliable publications and thus datasets that could form the basis of further AI research. This review shows a need to create guideline-compliant datasets with a transparent, fully reported methodology and limited RoB.

Models able to accurately recognise emotions using physiological parameters can contribute to the development of many disciplines. They create the possibility of reaching more advanced levels of HCI, where a computer (or system, in general) can modify its behaviour depending on the identified interlocutor’s state and choose the reaction closest to natural social schemes [112].

While using wearable devices, users might be supported in maintaining a psychological and healthy life balance, e.g., by identifying sources of stress, anxiety, or tension during their everyday activities and receiving feedback about their organisms reactions and resources [113]. Furthermore, assessments made on the basis of their CV signals can be used to investigate the impact of different emotional and affective states on the risk of developing CV diseases [114].

Well-validated AI models can significantly support research in the field of health and medical sciences and emotion theory by facilitating the simple, quick, and more matter-of-fact evaluation of emotions and other states and, therefore, reducing the RoB resulting from participants’ incorrect reporting.

Among the implications of our study, we should first include the recommendation to incorporate current, reliable guidelines and standards in the methodology development process and use quality assessment and reporting tools, as this translates into more reliable data, which may result in developing better recognition models [32]. For primary studies, we suggest following the proposed checklist for RoB (see Appendix C) or other available tools, e.g., [32].

### Strengths and Limitations

The performed review has high standards [32,60,61,115]. The research question was precisely defined. We utilised multiple resources for collecting studies mentioned in Section 2. Inclusion and exclusion criteria were firstly discussed and recorded. Researchers who participated in this review have knowledge in multiple disciplines: computer science, psychology, HCI, medicine, and methodology. To ensure transparency, we provide all necessary information in the Appendices and Supplements with a permanent DOI [64].

On the other hand, we did not search any Chinese databases. Considering the growing amount of evidence in this language, we might not have considered a large amount of evidence and thus weakened our conclusions. Moreover, the use of the search strategy itself and the stages of identifying articles based on titles and abstracts may be a limitation. Due to such action, we may miss an extraordinary piece of work that did not meet our criteria due to its original form.

## 5. Conclusions

This paper systematically reviewed the datasets that include CV signals for AAER with AI methods and assessed their quality.

Due to poor reporting and not following methodological guidelines, the evidence, however, is limited. Nevertheless, according to our review, the most up-to-standards research was proposed by Correa et al. [78] and Marin et al. [85].

In the future, more attention should be put into controlling bias in research to ensure incremental knowledge gain. The quality of papers and reporting needs to be improved in order to propose and develop models that do not introduce biases. Preferably, authors should focus more on methodology and describe procedures thoroughly. We recommend following standardised guidelines of reporting [116].

Our next steps include the synthesis of gathered evidence with other physiological signals. Furthermore, we want to propose our own unbiased dataset for AAER for public use. Based on these data, we plan to improve our affective games [117,118,119].

## Figures and Tables

**Table 1:** Characteristics of stimuli in 18 included studies (21 experiments).

Variable (No. of Experiments Available for Calculations)		No (%) Mean (Range)
Type of stimuli (21)		
	Video (music, movie, ads)	11 (52.38)
	Audio (music excerpts)	4 (19.05)
	Game (FIFA 2016, Maze-Ball)	2 (9.52)
	Virtual Reality (videos, scenes)	2 (9.52)
	Self elicitation (actors)	1 (4.76)
	Mixed (TSST ^1^, video and meditation in one experiment)	1 (4.76)
Length of stimuli [seconds] (17)		304.60 (32–1200)
No. of stimuli in dataset (20)		27.70 (4–144)
No. of elicited emotions [classes] (18)		6.06 (3–23)

^1^ Trier Social Stress Test.

**Table 2:** Characteristics of devices and signals in 18 included studies (21 experiments).

Variable (No. of Datasets Available for Calculations) ^1^		No (%) Mean (Range)
Used devices (16)		
	Shimmer 2R	3 (18.75)
	BIOPAC MP150	3 (18.75)
	Biosemi ActiveTwo	2 (12.50)
	NeXus-10	1 (6.25)
	ProComp Infiniti	1 (6.25)
	BIOPAC BioNomadix	1 (6.25)
	BItalino	1 (6.25)
	RespiBAN Professional	1 (6.25)
	B-Alert x10	1 (6.25)
	Empatica E4	1 (6.25)
	Polar H7	1 (6.25)
	Zephyr BioHarness	1 (6.25)
	IOM Biofeedback	1 (6.25)
CV ^2^ signals recorded (18)		
	ECG ^3^	15 (83.33)
	HR ^4^	3 (16.67)
	BVP ^5^	3 (16.67)
	PPG ^6^	1 (5.56)
Sampling frequency [Hz] (12)		543.31 (32–2048)
Length of baseline recording [seconds] (7)		292.14 (5–1200)

^1^ some studies used more than one device or cardiovascular signal; ^2^ cardiovascular; ^3^ electrocardiogram; ^4^ heart rate; ^5^ blood volume pressure; ^6^ photoplethysmogram.

**Table 3:** Characteristics of population in 18 included studies (21 experiments).

Variable (No. of Experiments Available for Calculations)	No. (%) Mean (Range)
Participating people (21)	916 43.62 (3–250)
Eligible people (20)	812 40.60 (3–250)
Age (18)	23.8 (17–47)
Percentage of females (16)	45.13 (0–86)
Ethnicity (4)	
Chinese	2 (9.52)
European	2 (9.52)

**Table 4:** Risk of Bias (RoB) among 18 included studies.

Study ID	RoB Item ^1^								Overall Quality ^2^	
	1	2	3	4	5	6	7	8	Scenario 1	Scenario 2
[75]	NR	PY	PY	Y	Y	Y	Y	PN	Low	Low
[76]	NR	PY	PY	Y	PN	Y	Y	NR	Low	Low
[77]	NR	NR	NR	Y	PN	Y	Y	PN	Low	Low
[78]	NR	Y	PY	Y	Y	Y	Y	PY	Unclear	High
[79]	NR	PY	PN	PY	PN	Y	Y	N	Low	Low
[80]	NR	PN	N	PN	PN	N	N	PY	Low	Low
[81]	NR	Y	PN	PY	Y	Y	Y	PN	Low	Low
[82]	NR	Y	Y	PY	Y	Y	Y	PN	Low	Low
[83]	NR	NR	NR	PN	PN	Y	Y	NR	Low	Low
[84]	NR	NR	NR	NR	PN	Y	Y	N	Low	Low
[85]	NR	Y	Y	Y	Y	Y	Y	Y	Unclear	High
[86]	NR	PY	PY	NR	Y	Y	Y	NR	Unclear	Unclear
[87]	NR	Y	Y	NR	Y	Y	N	NR	Low	Low
[88]	NR	Y	Y	Y	Y	Y	PN	Y	Low	Low
[89]	NR	Y	PN	NR	PN	Y	Y	NR	Low	Low
[90]	NR	PY	PY	PY	Y	Y	Y	NR	Unclear	Unclear
[91]	NR	PN	PY	NR	Y	Y	Y	NR	Low	Low
[92]	NR	Y	NR	Y	Y	NR	N	NR	Low	Low

^1^ Y, PY, NR, PN, N stands for yes, partial yes, not reported, partial no, no; ^2^ for more details, see Appendix C.

## Data Availability

The data presented in this study are available on request from the corresponding author.

## Supplementary Materials

The following supporting information can be downloaded at: https://osf.io/kzj8y/ (accessed on 15 February 2022) in *Sensors* folder: Protocol; Supplementary File S1—list of all included studies.

## Abbreviations

The following abbreviations are used in this manuscript:

AAER	automated affect and/or emotion recognition
ABP	arterial blood pressure
AI	artificial intelligence
AMIGOS	a dataset for Affect, personality and Mood research on Individuals and GrOupS
ASCERTAIN	a multimodal databaASe for impliCit pERsonaliTy and Affect recognitIoN using commercial physiological sensors
AuDB-4	AUgsburg Database of Biosignal 4
BVP	blood volume pressure
CV	cardiovascular
DAG	Database for Affective Gaming
DECAF	MEG-based multimodal database for DECoding AFfective physiological responses
DEAP	Database for Emotion Analysis using Physiological signals
DL	deep learning
DTA	diagnostic test accuracy
ECG	electrocardiogram
EDA	electrodermal activity
EEG	electroencephalography
EMG	electromyography
emoFBVP	database of multimodal (Face, Body gesture, Voice and Physiological signals) recordings
EPQ	Eysenck Personality Questionnaire
ERS	Emotion Recognition Smartwatch
HCI	human–computer interaction
HR	heart rate
HRV	heart rate variability
ICP	intracranial pressure
ITMDER	IT Multimodal Dataset for Emotion Recognition
MAHNOB-HCI	Multimodal Analysis of Human NOnverbal Behaviour in real-world settings-Human-Computer Interaction
MAPD	Multi-subject Affective Physiological Database
MD	Mazeball Dataset
MEG	magnetoencephalography
ML	machine learning
MPED	Multi-modal Physiological Emotion Database for discrete emotion recognition
NB	naive Bayes
OSF	Open Science Framework
PANAS	Positive and Negative Affect Schedule
POX	pulse oximetry
PPG	photoplethysmogram
PPV	pulse pressure variation
PRISMA	Preferred Reporting Items for Systematic reviews and Meta-Analyses
PROBAST	Prediction model Risk Of Bias ASsessment Tool
QUADAS	QUality Assessment of Diagnostic Accuracy Studies
RF	random forest
RoB	Risk of Bias
SAM	Self-Assessment Manikin
TSST	Trier Social Stress Test
VRAD	Virtual Reality Affective Dataset
WESAD	WEarable Stress and Affect Detection

## Appendix A. Papers Included in This Focused Review

The following papers were included in this focused review: [75,76,77,78,79,80,81,82,83,84,85,86,87,88,89,90,91,92].

## Appendix B. Papers Excluded

**Table A1:** Excluded Studies.

Study ID	Reason of Excluding
[120]	Wrong study design
[121]	Wrong study design
[122]	Wrong study design
[123]	Wrong study design
[124]	Wrong study design
[125]	Wrong study design
[126]	Wrong study design
[127]	Wrong study design
[128]	Wrong study design
[129]	Wrong study design
[130]	Wrong study design
[131]	Wrong study design
[132]	Wrong study design
[133]	Wrong study design
[134]	Wrong study design
[135]	Wrong study design
[136]	Wrong population
[137]	Wrong population
[138]	Wrong population
[139]	Wrong target
[140]	Wrong target
[141]	Wrong target
[142]	Wrong target
[143]	Wrong target
[144]	Wrong target
[145]	Wrong target
[146]	Wrong index method
[147]	Wrong index method
[148]	Wrong type of data
[149]	Wrong setting
[150]	Wrong setting
[151]	Wrong setting
[152]	Wrong outcomes
[153]	Wrong outcomes
[154]	Wrong outcomes
[155]	Wrong outcomes
[156]	Wrong outcomes
[157]	Wrong outcomes

## Appendix C. Risk of Bias Tool

**Table A2:** Risk of bias tool.

Domain ^1^	Review Authors’s Judgement	Criteria for Judgement	
Sample [74]	1. Was the sample size prespecified?	Yes/partial yes	The experiment was preceded by calculating the minimum sample size, and the method used was adequate and well-described.
		No/partial no	It is stated that the minimum sample size has not been calculated, or it has been calculated, but no details of the method used are provided.
		Not reported	No sufficient information is provided in this regard.
Sample [74]	2. Were eligibility criteria for the experiment provided?	Yes/partial yes	The criteria for inclusion in the experiment are specified.
		No/partial no	The criteria for inclusion in the experiment were used, however not specified in the article.
		Not reported	No sufficient information is provided in this regard.
Participants [73]	3. Were all inclusions and exclusions of participants appropriate?	Yes/partial yes	The criteria for inclusion and exclusion are relevant to the aim of the study. Conditions that may affect the participant’s state or collected physiological signals and ability to recognise emotions were considered, including cardiovascular and mental disorders.
		No/partial no	The established criteria for inclusion and exclusion are irrelevant to the aim of the study.
		Not reported	No sufficient information is provided in this regard.
Measurement [74]	4. Was the measurement of exposition clearly stated?	Yes/partial yes	The selection of stimuli is adequately justified in the context of eliciting emotions, e.g., selection from a standardised database, pilot studies.
		No/partial no	The selection of stimuli was carried out based on inadequate criteria.
		Not reported	No sufficient information is provided in this regard.
Measurement [74]	5. Was the measurement of outcome clearly stated?	Yes/partial yes	The assessment tool used for emotions measurement is described in detail, adequate, and validated.
		No/partial no	The assessment tool used for emotions measurement is not described, or the measurement method is inadequate, or not validated.
		Not reported	No sufficient information is provided in this regard.
Flow and Timing [72]	6. Did all participants receive a reference standard?	Yes/partial yes	Emotions were measured in all participants, and the measurement was performed after each stimulus.
		No/partial no	Not all participants had their emotions measured.
		Not reported	No sufficient information is provided in this regard.
Flow and Timing [72]	7. Did participants receive the same reference standard?	Yes/partial yes	The same assessment standard was used in all participants who had their emotions measured
		No/partial no	A different assessment standard was used in some of the participants to measure their emotions.
		Not reported	No sufficient information is provided in this regard.
Control of confounders [74]	8. Were the confounders measured?	Yes/partial yes	Adequate confounding factors were measured, and relevant justification is provided.
		No/partial no	The control of confounding factors is not justified, or the measured factors are inadequate.
		Not reported	No sufficient information is provided in regard to confounding factors.
**Scenario 1**: Overall quality (elicitation) **Scenario 2**: Overall quality (without judgement of 1. item)		High	All judgements are yes or partial yes.
		Low	At least one judgement is no or partial no.
		Unclear	All judgements are yes or partial yes with at least one not reported.

^1^ the specific domain was based on an instrument provided in the reference.

## References

[1] Hacker P. (2018). Teaching fairness to artificial intelligence: Existing and novel strategies against algorithmic discrimination under EU law. Common Mark. Law Rev..

[2] Butterworth M. (2018). The ICO and artificial intelligence: The role of fairness in the GDPR framework. Comput. Law Secur. Rev..

[3] Fan X., Yan Y., Wang X., Yan H., Li Y., Xie L., Yin E. Emotion Recognition Measurement based on Physiological Signals. Proceedings of the 2020 13th International Symposium on Computational Intelligence and Design (ISCID).

[4] Xia H., Wu J., Shen X., Yang F. The Application of Artificial Intelligence in Emotion Recognition. Proceedings of the 2020 International Conference on Intelligent Computing and Human-Computer Interaction (ICHCI).

[5] Jemioło P., Storman D., Giżycka B., Ligęza A. (2021). Emotion elicitation with stimuli datasets in automatic affect recognition studies—Umbrella review. Proceedings of the IFIP Conference on Human-Computer Interaction.

[6] Ekman P., Friesen W.V., O’sullivan M., Chan A., Diacoyanni-Tarlatzis I., Heider K., Krause R., LeCompte W.A., Pitcairn T., Ricci-Bitti P.E. (1987). Universals and cultural differences in the judgments of facial expressions of emotion. J. Personal. Soc. Psychol..

[7] Barrett L.F. (2017). How Emotions Are Made: The Secret Life of the Brain.

[8] Plutchik R. (1980). A general psychoevolutionary theory of emotion. Theories of Emotion.

[9] Sarma P., Barma S. (2020). Review on Stimuli Presentation for Affect Analysis Based on EEG. IEEE Access.

[10] Bradley M.M., Lang P.J. (1994). Measuring emotion: The self-assessment manikin and the semantic differential. J. Behav. Ther. Exp. Psychiatry.

[11] Bandara D., Song S., Hirshfield L., Velipasalar S. (2016). A more complete picture of emotion using electrocardiogram and electrodermal activity to complement cognitive data. Proceedings of the International Conference on Augmented Cognition.

[12] Nardelli M., Greco A., Valenza G., Lanata A., Bailón R., Scilingo E.P. A novel heart rate variability analysis using lagged poincaré plot: A study on hedonic visual elicitation. Proceedings of the 2017 39th Annual International Conference of the IEEE Engineering in Medicine and Biology Society (EMBC).

[13] Jang E.H., Park B.J., Kim S.H., Chung M.A., Park M.S., Sohn J.H. Emotion classification based on bio-signals emotion recognition using machine learning algorithms. Proceedings of the 2014 International Conference on Information Science, Electronics and Electrical Engineering.

[14] Kołakowska A., Szwoch W., Szwoch M. (2020). A review of emotion recognition methods based on data acquired via smartphone sensors. Sensors.

[15] Zhao B., Wang Z., Yu Z., Guo B. EmotionSense: Emotion recognition based on wearable wristband. Proceedings of the 2018 IEEE SmartWorld, Ubiquitous Intelligence & Computing, Advanced & Trusted Computing, Scalable Computing & Communications, Cloud & Big Data Computing, Internet of People and Smart City Innovation (SmartWorld/SCALCOM/UIC/ATC/CBDCom/IOP/SCI).

[16] Akalin N., Köse H. Emotion recognition in valence-arousal scale by using physiological signals. Proceedings of the 2018 26th Signal Processing and Communications Applications Conference (SIU).

[17] Nasoz F., Lisetti C.L., Vasilakos A.V. (2010). Affectively intelligent and adaptive car interfaces. Inf. Sci..

[18] Hsiao P.W., Chen C.P. Effective attention mechanism in dynamic models for speech emotion recognition. Proceedings of the 2018 IEEE International Conference on Acoustics, Speech and Signal Processing (ICASSP).

[19] Shu L., Yu Y., Chen W., Hua H., Li Q., Jin J., Xu X. (2020). Wearable emotion recognition using heart rate data from a smart bracelet. Sensors.

[20] Ragot M., Martin N., Em S., Pallamin N., Diverrez J.M. (2017). Emotion recognition using physiological signals: Laboratory vs. wearable sensors. In Proceedings of the International Conference on Applied Human Factors and Ergonomics.

[21] Ali H., Hariharan M., Yaacob S., Adom A.H. (2015). Facial emotion recognition using empirical mode decomposition. Expert Syst. Appl..

[22] Liu Z.T., Wu M., Cao W.H., Mao J.W., Xu J.P., Tan G.Z. (2018). Speech emotion recognition based on feature selection and extreme learning machine decision tree. Neurocomputing.

[23] Gómez-Zaragozá L., Marín-Morales J., Parra E., Guixeres J., Alcañiz M. (2020). Speech Emotion Recognition from Social Media Voice Messages Recorded in the Wild. Proceedings of the International Conference on Human-Computer Interaction.

[24] Abdullah S.M.S.A., Ameen S.Y.A., Sadeeq M.A., Zeebaree S. (2021). Multimodal emotion recognition using deep learning. J. Appl. Sci. Technol. Trends.

[25] Harper R., Southern J. (2020). A bayesian deep learning framework for end-to-end prediction of emotion from heartbeat. IEEE Trans. Affect. Comput..

[26] Oh S., Lee J.Y., Kim D.K. (2020). The design of CNN architectures for optimal six basic emotion classification using multiple physiological signals. Sensors.

[27] Ravindran A.S., Nakagome S., Wickramasuriya D.S., Contreras-Vidal J.L., Faghih R.T. Emotion recognition by point process characterization of heartbeat dynamics. Proceedings of the 2019 IEEE Healthcare Innovations and Point of Care Technologies, (HI-POCT).

[28] Gadea G.H., Kreuder A., Stahlschmidt C., Schnieder S., Krajewski J. (2018). Brute Force ECG Feature Extraction Applied on Discomfort Detection. Proceedings of the International Conference on Information Technologies in Biomedicine.

[29] Moharreri S., Dabanloo N.J., Maghooli K. (2019). Detection of emotions induced by colors in compare of two nonlinear mapping of heart rate variability signal: Triangle and parabolic phase space (TPSM, PPSM). J. Med. Biol. Eng..

[30] Basu A., Routray A., Shit S., Deb A.K. Human emotion recognition from facial thermal image based on fused statistical feature and multi-class SVM. Proceedings of the 2015 Annual IEEE India Conference (INDICON).

[31] Ferdinando H., Seppänen T., Alasaarela E. (2017). Emotion recognition using neighborhood components analysis and ecg/hrv-based features. Proceedings of the International Conference on Pattern Recognition Applications and Methods.

[32] Higgins J.P., Thomas J., Chandler J., Cumpston M., Li T., Page M.J., Welch V.A. (2019). Cochrane Handbook for Systematic Reviews of Interventions.

[33] Mamica M., Kapłon P., Jemioło P. (2021). EEG-Based Emotion Recognition Using Convolutional Neural Networks. Proceedings of the International Conference on Conceptual Structures.

[34] Saxena A., Khanna A., Gupta D. (2020). Emotion recognition and detection methods: A comprehensive survey. J. Artif. Intell. Syst..

[35] Resnick B. More Social Science Studies just Failed to Replicate. Here’s Why This Is Good. Https://www.vox.com/science-and-health/2018/8/27/17761466/psychology-replication-crisis-nature-social-science.

[36] Maxwell S.E., Lau M.Y., Howard G.S. (2015). Is psychology suffering from a replication crisis? What does “failure to replicate” really mean?. Am. Psychol..

[37] Kilkenny M.F., Robinson K.M. (2018). Data Quality: “Garbage In—Garbage Out”. Health Inf. Manag. J..

[38] Vidgen B., Derczynski L. (2020). Directions in abusive language training data, a systematic review: Garbage in, garbage out. PLoS ONE.

[39] Stodden V., Seiler J., Ma Z. (2018). An empirical analysis of journal policy effectiveness for computational reproducibility. Proc. Natl. Acad. Sci. USA.

[40] Fehr J., Heiland J., Himpe C., Saak J. (2016). Best practices for replicability, reproducibility and reusability of computer-based experiments exemplified by model reduction software. arXiv.

[41] Mann F., von Walter B., Hess T., Wigand R.T. (2009). Open access publishing in science. Commun. ACM.

[42] Kluyver T., Ragan-Kelley B., Pérez F., Granger B.E., Bussonnier M., Frederic J., Kelley K., Hamrick J.B., Grout J., Corlay S. (2016). Jupyter Notebooks—A Publishing Format for Reproducible Computational Workflows.

[43] ReScicenceX Http://rescience.org/x.

[44] ReScicence C Https://rescience.github.io/.

[45] Simmons J.P., Nelson L.D., Simonsohn U. (2021). Pre-registration: Why and how. J. Consum. Psychol..

[46] Nosek B.A., Ebersole C.R., DeHaven A.C., Mellor D.T. (2018). The preregistration revolution. Proc. Natl. Acad. Sci. USA.

[47] Joffe M.M., Ten Have T.R., Feldman H.I., Kimmel S.E. (2004). Model selection, confounder control, and marginal structural models: Review and new applications. Am. Stat..

[48] Pourhoseingholi M.A., Baghestani A.R., Vahedi M. (2012). How to control confounding effects by statistical analysis. Gastroenterol. Hepatol. Bed Bench.

[49] Salminen J.K., Saarijärvi S., Äärelä E., Toikka T., Kauhanen J. (1999). Prevalence of alexithymia and its association with sociodemographic variables in the general population of Finland. J. Psychosom. Res..

[50] Greenaway K.H., Kalokerinos E.K., Williams L.A. (2018). Context is everything (in emotion research). Soc. Personal. Psychol. Compass.

[51] Saganowski S., Dutkowiak A., Dziadek A., Dzieżyc M., Komoszyńska J., Michalska W., Polak A., Ujma M., Kazienko P. Emotion recognition using wearables: A systematic literature review-work-in-progress. Proceedings of the 2020 IEEE International Conference on Pervasive Computing and Communications Workshops (PerCom Workshops).

[52] Peake J.M., Kerr G., Sullivan J.P. (2018). A critical review of consumer wearables, mobile applications, and equipment for providing biofeedback, monitoring stress, and sleep in physically active populations. Front. Physiol..

[53] Shu L., Xie J., Yang M., Li Z., Li Z., Liao D., Xu X., Yang X. (2018). A review of emotion recognition using physiological signals. Sensors.

[54] Kutt K., Nalepa G.J., Giżycka B., Jemiolo P., Adamczyk M. Bandreader-a mobile application for data acquisition from wearable devices in affective computing experiments. Proceedings of the 2018 11th International Conference on Human System Interaction (HSI).

[55] Vallejo-Correa P., Monsalve-Pulido J., Tabares-Betancur M. (2021). A systematic mapping review of context-aware analysis and its approach to mobile learning and ubiquitous learning processes. Comput. Sci. Rev..

[56] Bardram J.E., Matic A. (2020). A decade of ubiquitous computing research in mental health. IEEE Pervasive Comput..

[57] Cárdenas-Robledo L.A., Peña-Ayala A. (2018). Ubiquitous learning: A systematic review. Telemat. Inform..

[58] Paré G., Kitsiou S. (2017). Methods for literature reviews. Handbook of eHealth Evaluation: An Evidence-Based Approach [Internet].

[59] Okoli C. (2015). A guide to conducting a standalone systematic literature review. Commun. Assoc. Inf. Syst..

[60] Moher D., Liberati A., Tetzlaff J., Altman D.G. (2009). PRISMA 2009 flow diagram. PRISMA Statement.

[61] Liberati A., Altman D., Tetzlaff J., Mulrow C., Gøtzsche P., Ioannidis J., Clarke M., Devereaux P. (2009). The PRISMA statement for reporting systematic and meta-analyses of studies that evaluate interventions. PLoS Med..

[62] Jemioło P., Storman D., Mamica M., Szymkowski M., Orzechowski P., Dranka W. Emotion Recognition from Cardiovascular Signals Using Artificial Intelligence—A Systematic Review. Https://osf.io/nj7ut.

[63] Jemioło P., Storman D., Mamica M., Szymkowski M., Orzechowski P. (2022). Automated Affect and Emotion Recognition from Cardiovascular Signals—A Systematic Overview of the Field. Proceedings of the Hawaii International Conference on System Sciences.

[64] Jemioło P., Storman D., Mamica M., Szymkowski M., Orzechowski P., Dranka W. Emotion Recognition from Cardiovascular Signals Using Artificial Intelligence—A Systematic Review. Https://osf.io/kzj8y/.

[65] Konar A., Chakraborty A. (2015). Emotion Recognition: A Pattern Analysis Approach.

[66] Kim K.H., Bang S.W., Kim S.R. (2004). Emotion recognition system using short-term monitoring of physiological signals. Med. Biol. Eng. Comput..

[67] Copeland B. Artificial Intelligence: Definition, Examples, and Applications. Https://www.britannica.com/technology/artificial-intelligence.

[68] Craik A., He Y., Contreras-Vidal J.L. (2019). Deep learning for electroencephalogram (EEG) classification tasks: A review. J. Neural Eng..

[69] Botchkarev A. (2018). Performance metrics (error measures) in machine learning regression, forecasting and prognostics: Properties and typology. arXiv.

[70] McNames J., Aboy M. (2007). Statistical modeling of cardiovascular signals and parameter estimation based on the extended Kalman filter. IEEE Trans. Biomed. Eng..

[71] Ouzzani M., Hammady H., Fedorowicz Z., Elmagarmid A. (2016). Rayyan—A web and mobile app for systematic reviews. Syst. Rev..

[72] Whiting P.F., Rutjes A.W., Westwood M.E., Mallett S., Deeks J.J., Reitsma J.B., Leeflang M.M., Sterne J.A., Bossuyt P.M. (2011). QUADAS-2: A revised tool for the quality assessment of diagnostic accuracy studies. Ann. Intern. Med..

[73] Wolff R.F., Moons K.G., Riley R.D., Whiting P.F., Westwood M., Collins G.S., Reitsma J.B., Kleijnen J., Mallett S. (2019). PROBAST: A tool to assess the risk of bias and applicability of prediction model studies. Ann. Intern. Med..

[74] Benton M.J., Hutchins A.M., Dawes J.J. (2020). Effect of menstrual cycle on resting metabolism: A systematic review and meta-analysis. PLoS ONE.

[75] Koelstra S., Muhl C., Soleymani M., Lee J.S., Yazdani A., Ebrahimi T., Pun T., Nijholt A., Patras I. (2011). Deap: A database for emotion analysis; using physiological signals. IEEE Trans. Affect. Comput..

[76] Soleymani M., Lichtenauer J., Pun T., Pantic M. (2011). A multimodal database for affect recognition and implicit tagging. IEEE Trans. Affect. Comput..

[77] Abadi M.K., Subramanian R., Kia S.M., Avesani P., Patras I., Sebe N. (2015). DECAF: MEG-based multimodal database for decoding affective physiological responses. IEEE Trans. Affect. Comput..

[78] Correa J.A.M., Abadi M.K., Sebe N., Patras I. (2021). Amigos: A dataset for affect, personality and mood research on individuals and groups. IEEE Trans. Affect. Comput..

[79] Subramanian R., Wache J., Abadi M.K., Vieriu R.L., Winkler S., Sebe N. (2016). ASCERTAIN: Emotion and personality recognition using commercial sensors. IEEE Trans. Affect. Comput..

[80] Kim J., André E. (2008). Emotion recognition based on physiological changes in music listening. IEEE Trans. Pattern Anal. Mach. Intell..

[81] Quiroz J.C., Geangu E., Yong M.H. (2018). Emotion recognition using smart watch sensor data: Mixed-design study. JMIR Ment. Health.

[82] Pinto J. (2019). Exploring Physiological Multimodality for Emotional Assessment.

[83] Yang W., Rifqi M., Marsala C., Pinna A. Physiological-based emotion detection and recognition in a video game context. Proceedings of the 2018 International Joint Conference on Neural Networks (IJCNN).

[84] Gupta R., Khomami Abadi M., Cárdenes Cabré J.A., Morreale F., Falk T.H., Sebe N. A quality adaptive multimodal affect recognition system for user-centric multimedia indexing. Proceedings of the 2016 ACM on International Conference on Multimedia Retrieval.

[85] Marín-Morales J., Higuera-Trujillo J.L., Greco A., Guixeres J., Llinares C., Scilingo E.P., Alcañiz M., Valenza G. (2018). Affective computing ual reality: Emotion recognition from brain and heartbeat dynamics using wearable sensors. Sci. Rep..

[86] Hsu Y.L., Wang J.S., Chiang W.C., Hung C.H. (2017). Automatic ECG-based emotion recognition in music listening. IEEE Trans. Affect. Comput..

[87] Schmidt P., Reiss A., Duerichen R., Marberger C., Van Laerhoven K. Introducing wesad, a multimodal dataset for wearable stress and affect detection. Proceedings of the 20th ACM International Conference on Multimodal Interaction.

[88] Song T., Zheng W., Lu C., Zong Y., Zhang X., Cui Z. (2019). MPED: A multi-modal physiological emotion database for discrete emotion recognition. IEEE Access.

[89] Ranganathan H., Chakraborty S., Panchanathan S. Multimodal emotion recognition using deep learning architectures. Proceedings of the 2016 IEEE Winter Conference on Applications of Computer Vision (WACV).

[90] Katsigiannis S., Ramzan N. (2017). DREAMER: A database for emotion recognition through EEG and ECG signals from wireless low-cost off-the-shelf devices. IEEE J. Biomed. Health Inform..

[91] Huang W., Liu G., Wen W. MAPD: A Multi-subject Affective Physiological Database. Proceedings of the 2014 Seventh International Symposium on Computational Intelligence and Design.

[92] Yannakakis G.N., Martínez H.P., Jhala A. (2010). Towards affective camera control in games. User Model. User-Adapt. Interact..

[93] McInnes M.D., Moher D., Thombs B.D., McGrath T.A., Bossuyt P.M., Clifford T., Cohen J.F., Deeks J.J., Gatsonis C., Hooft L. (2018). Preferred reporting items for a systematic review and meta-analysis of diagnostic test accuracy studies: The PRISMA-DTA statement. JAMA.

[94] Wierzbicka A. (1992). Defining emotion concepts. Cogn. Sci..

[95] Wierzbicka A. (1994). Emotion, language, and cultural scripts. Emotion and Culture: Empirical Studies of Mutual Influence.

[96] Cook D.A., Levinson A.J., Garside S. (2011). Method and reporting quality in health professions education research: A systematic review. Med. Educ..

[97] Wijasena H.Z., Ferdiana R., Wibirama S. A Survey of Emotion Recognition using Physiological Signal in Wearable Devices. Proceedings of the 2021 International Conference on Artificial Intelligence and Mechatronics Systems (AIMS).

[98] Saganowski S., Kazienko P., Dziezyc M., Jakimow P., Komoszynska J., Michalska W., Dutkowiak A., Polak A., Dziadek A., Ujma M. Consumer Wearables and Affective Computing for Wellbeing Support. Proceedings of the MobiQuitous 2020—17th EAI International Conference on Mobile and Ubiquitous Systems: Computing, Networking and Services.

[99] Schmidt P., Reiss A., Dürichen R., Laerhoven K.V. (2019). Wearable-Based Affect Recognition—A Review. Sensors.

[100] Merone M., Soda P., Sansone M., Sansone C. (2017). ECG databases for biometric systems: A systematic review. Expert Syst. Appl..

[101] Da Silva H.P., Lourenço A., Fred A., Raposo N., Aires-de Sousa M. (2014). Check Your Biosignals Here: A new dataset for off-the-person ECG biometrics. Comput. Methods Programs Biomed..

[102] Hong S., Zhou Y., Shang J., Xiao C., Sun J. (2020). Opportunities and challenges of deep learning methods for electrocardiogram data: A systematic review. Comput. Biol. Med..

[103] Santamaria-Granados L., Munoz-Organero M., Ramirez-Gonzalez G., Abdulhay E., Arunkumar N. (2018). Using deep convolutional neural network for emotion detection on a physiological signals dataset (AMIGOS). IEEE Access.

[104] Soroush M.Z., Maghooli K., Setarehdan S.K., Nasrabadi A.M. (2017). A review on EEG signals based emotion recognition. Int. Clin. Neurosci. J..

[105] Suhaimi N.S., Mountstephens J., Teo J. (2020). EEG-based emotion recognition: A state-of-the-art review of current trends and opportunities. Comput. Intell. Neurosci..

[106] Wagh K.P., Vasanth K. (2019). Electroencephalograph (EEG) based emotion recognition system: A review. Innovations in Electronics and Communication Engineering.

[107] Egger M., Ley M., Hanke S. (2019). Emotion recognition from physiological signal analysis: A review. Electron. Notes Theor. Comput. Sci..

[108] Jerritta S., Murugappan M., Nagarajan R., Wan K. Physiological signals based human emotion recognition: A review. Proceedings of the 2011 IEEE 7th International Colloquium on Signal Processing and its Applications.

[109] Ali M., Mosa A.H., Al Machot F., Kyamakya K. (2018). Emotion recognition involving physiological and speech signals: A comprehensive review. Recent Advances in Nonlinear Dynamics and Synchronization.

[110] Szwoch W. Using physiological signals for emotion recognition. Proceedings of the 2013 6th International Conference on Human System Interactions (HSI).

[111] Callejas-Cuervo M., Martínez-Tejada L.A., Alarcón-Aldana A.C. (2017). Emotion recognition techniques using physiological signals and video games-Systematic review. Rev. Fac. Ing..

[112] Marechal C., Mikolajewski D., Tyburek K., Prokopowicz P., Bougueroua L., Ancourt C., Wegrzyn-Wolska K. (2018). Survey on AI-Based Multimodal Methods for Emotion Detection. High-Performance Modelling and Simulation for Big Data Application.

[113] Picard R.W. (1997). Affective Computing.

[114] Amira T., Dan I., Az-eddine B., Ngo H.H., Said G., Katarzyna W.W. Monitoring chronic disease at home using connected devices. Proceedings of the 2018 13th Annual Conference on System of Systems Engineering (SoSE).

[115] Khan K.S., Kunz R., Kleijnen J., Antes G. (2003). Five steps to conducting a systematic review. J. R. Soc. Med..

[116] Enhancing the Quality and Transparency of Health Research. Https://www.equator-network.org/.

[117] Giżycka B., Jemioło P., Domarecki S., Świder K., Wiśniewski M., Mielczarek Ł. (2019). A Thin Light Blue Line—Towards Balancing Educational and Recreational Values of Serious Games. In Proceedings of the 3rd Workshop on Affective Computing and Context Awareness in Ambient Intelligence.

[118] Jemioło P., Giżycka B., Nalepa G.J. (2019). Prototypes of arcade games enabling affective interaction. Proceedings of the International Conference on Artificial Intelligence and Soft Computing.

[119] Nalepa G.J., Kutt K., Giżycka B., Jemioło P., Bobek S. (2019). Analysis and use of the emotional context with wearable devices for games and intelligent assistants. Sensors.

[120] Benovoy M., Cooperstock J.R., Deitcher J. Biosignals analysis and its application in a performance setting. Proceedings of the International Conference on Bio-Inspired Systems and Signal Processing.

[121] Mera K., Ichimura T. (2004). Emotion analyzing method using physiological state. Proceedings of the International Conference on Knowledge-Based and Intelligent Information and Engineering Systems.

[122] Wang Y., Mo J. Emotion feature selection from physiological signals using tabu search. Proceedings of the 2013 25th Chinese Control and Decision Conference (CCDC).

[123] Guendil Z., Lachiri Z., Maaoui C., Pruski A. Emotion recognition from physiological signals using fusion of wavelet based features. Proceedings of the 2015 7th International Conference on Modelling, Identification and Control (ICMIC).

[124] Zong C., Chetouani M. Hilbert-Huang transform based physiological signals analysis for emotion recognition. Proceedings of the 2009 IEEE International Symposium on Signal Processing and Information Technology (ISSPIT).

[125] Joesph C., Rajeswari A., Premalatha B., Balapriya C. Implementation of physiological signal based emotion recognition algorithm. Proceedings of the 2020 IEEE 36th International Conference on Data Engineering (ICDE).

[126] Leon E., Clarke G., Sepulveda F., Callaghan V. Neural network-based improvement in class separation of physiological signals for emotion classification. Proceedings of the IEEE Conference on Cybernetics and Intelligent Systems.

[127] Siow S.C., Loo C.K., Tan A.W., Liew W.S. Adaptive Resonance Associative Memory for multi-channel emotion recognition. Proceedings of the 2010 IEEE EMBS Conference on Biomedical Engineering and Sciences (IECBES).

[128] Perez-Rosero M.S., Rezaei B., Akcakaya M., Ostadabbas S. Decoding emotional experiences through physiological signal processing. Proceedings of the 2017 IEEE International Conference on Acoustics, Speech and Signal Processing (ICASSP).

[129] Sokolova M.V., Fernández-Caballero A., López M.T., Martínez-Rodrigo A., Zangróniz R., Pastor J.M. (2015). A distributed architecture for multimodal emotion identification. Proceedings of the 13th International Conference on Practical Applications of Agents and Multi-Agent Systems.

[130] Shirahama K., Grzegorzek M. (2016). Emotion recognition based on physiological sensor data using codebook approach. Proceedings of the Conference of Information Technologies in Biomedicine.

[131] Gong P., Ma H.T., Wang Y. Emotion recognition based on the multiple physiological signals. Proceedings of the 2016 IEEE International Conference on Real-time Computing and Robotics (RCAR).

[132] Jain M., Saini S., Kant V. A hybrid approach to emotion recognition system using multi-discriminant analysis & k-nearest neighbour. Proceedings of the 2017 International Conference on Advances in Computing, Communications and Informatics (ICACCI).

[133] Guendil Z., Lachiri Z., Maaoui C., Pruski A. Multiresolution framework for emotion sensing in physiological signals. Proceedings of the 2016 2nd International Conference on Advanced Technologies for Signal and Image Processing (ATSIP).

[134] Wong W.M., Tan A.W., Loo C.K., Liew W.S. PSO optimization of synergetic neural classifier for multichannel emotion recognition. Proceedings of the 2010 Second World Congress on Nature and Biologically Inspired Computing (NaBIC).

[135] Guo X. (2011). Study of emotion recognition based on electrocardiogram and RBF neural network. Procedia Eng..

[136] Zhu H., Han G., Shu L., Zhao H. (2020). ArvaNet: Deep Recurrent Architecture for PPG-Based Negative Mental-State Monitoring. IEEE Trans. Comput. Soc. Syst..

[137] Wu C.H., Kuo B.C., Tzeng G.H. (2014). Factor analysis as the feature selection method in an Emotion Norm Database. Proceedings of the Asian Conference on Intelligent Information and Database Systems.

[138] Akbulut F.P., Perros H.G., Shahzad M. (2020). Bimodal affect recognition based on autoregressive hidden Markov models from physiological signals. Comput. Methods Programs Biomed..

[139] Takahashi M., Kubo O., Kitamura M., Yoshikawa H. Neural network for human cognitive state estimation. Proceedings of the IEEE/RSJ International Conference on Intelligent Robots and Systems (IROS’94).

[140] Gao Y., Barreto A., Adjouadi M. (2010). An affective sensing approach through pupil diameter processing and SVM classification. Biomed. Sci. Instrum..

[141] Mohino-Herranz I., Gil-Pita R., Rosa-Zurera M., Seoane F. (2019). Activity recognition using wearable physiological measurements: Selection of features from a comprehensive literature study. Sensors.

[142] Bonarini A., Costa F., Garbarino M., Matteucci M., Romero M., Tognetti S. (2011). Affective videogames: The problem of wearability and comfort. Proceedings of the International Conference on Human-Computer Interaction.

[143] Alqahtani F., Katsigiannis S., Ramzan N. ECG-based affective computing for difficulty level prediction in intelligent tutoring systems. Proceedings of the 2019 UK/China Emerging Technologies (UCET).

[144] Xu J., Hu Z., Zou J., Bi A. (2019). Intelligent emotion detection method based on deep learning in medical and health data. IEEE Access.

[145] Wendt C., Popp M., Karg M., Kuhnlenz K. Physiology and HRI: Recognition of over-and underchallenge. Proceedings of the RO-MAN 2008—The 17th IEEE International Symposium on Robot and Human Interactive Communication.

[146] Omata M., Moriwaki K., Mao X., Kanuka D., Imamiya A. Affective rendering: Visual effect animations for affecting user arousal. Proceedings of the 2012 International Conference on Multimedia Computing and Systems.

[147] van den Broek E.L., Schut M.H., Westerink J.H., Tuinenbreijer K. (2009). Unobtrusive sensing of emotions (USE). J. Ambient Intell. Smart Environ..

[148] Quiroz J.C., Yong M.H., Geangu E. Emotion-recognition using smart watch accelerometer data: Preliminary findings. Proceedings of the 2017 ACM International Joint Conference on Pervasive and Ubiquitous Computing.

[149] Althobaiti T., Katsigiannis S., West D., Bronte-Stewart M., Ramzan N. Affect detection for human-horse interaction. Proceedings of the 2018 21st Saudi Computer Society National Computer Conference (NCC).

[150] Dobbins C., Fairclough S. Detecting negative emotions during real-life driving via dynamically labelled physiological data. Proceedings of the 2018 IEEE International Conference on Pervasive Computing and Communications Workshops (PerCom Workshops).

[151] Jo Y., Lee H., Cho A., Whang M. (2017). Emotion Recognition Through Cardiovascular Response in Daily Life Using KNN Classifier. Advances in Computer Science and Ubiquitous Computing.

[152] Hamdi H., Richard P., Suteau A., Allain P. Emotion assessment for affective computing based on physiological responses. Proceedings of the 2012 IEEE International Conference on Fuzzy Systems.

[153] Moghimi S., Chau T., Guerguerian A.M. Using prefrontal cortex near-infrared spectroscopy and autonomic nervous system activity for identifying music-induced emotions. Proceedings of the 2013 6th International IEEE/EMBS Conference on Neural Engineering (NER).

[154] Zhang Z., Tanaka E. Affective computing using clustering method for mapping human’s emotion. Proceedings of the 2017 IEEE International Conference on Advanced Intelligent Mechatronics (AIM).

[155] Reinerman-Jones L., Taylor G., Cosenzo K., Lackey S. (2011). Analysis of multiple physiological sensor data. Proceedings of the International Conference on Foundations of Augmented Cognition.

[156] Roza V., Postolache O., Groza V., Pereira J.D. Emotions Assessment on Simulated Flights. Proceedings of the 2019 IEEE International Symposium on Medical Measurements and Applications (MeMeA).

[157] Savran A., Ciftci K., Chanel G., Mota J., Viet L., Sankur B., Akarun L., Caplier A., Rombaut M. Emotion detection in the loop from brain signals and facial images. Proceedings of the eNTERFACE 2006 Workshop.

